# Supplementary material for: Differences of Rainfall–Malaria Associations in Lowland and Highland in Western Kenya
Source: Int J Environ Res Public Health. 2019 Sep 30;16(19):3693. doi: 10.3390/ijerph16193693 (PMC6801446; doi:10.3390/ijerph16193693)
Supplement: Supplementary file 1 [file ijerph-16-03693-s001.pdf]

Supplementary Materials

Differences of rainfall-malaria associations in lowland and highland in Western Kenya

Figure S1 monthly time series plots of the other hospitals (Kendu Bay, Maseno, Kericho and Kapsabet)

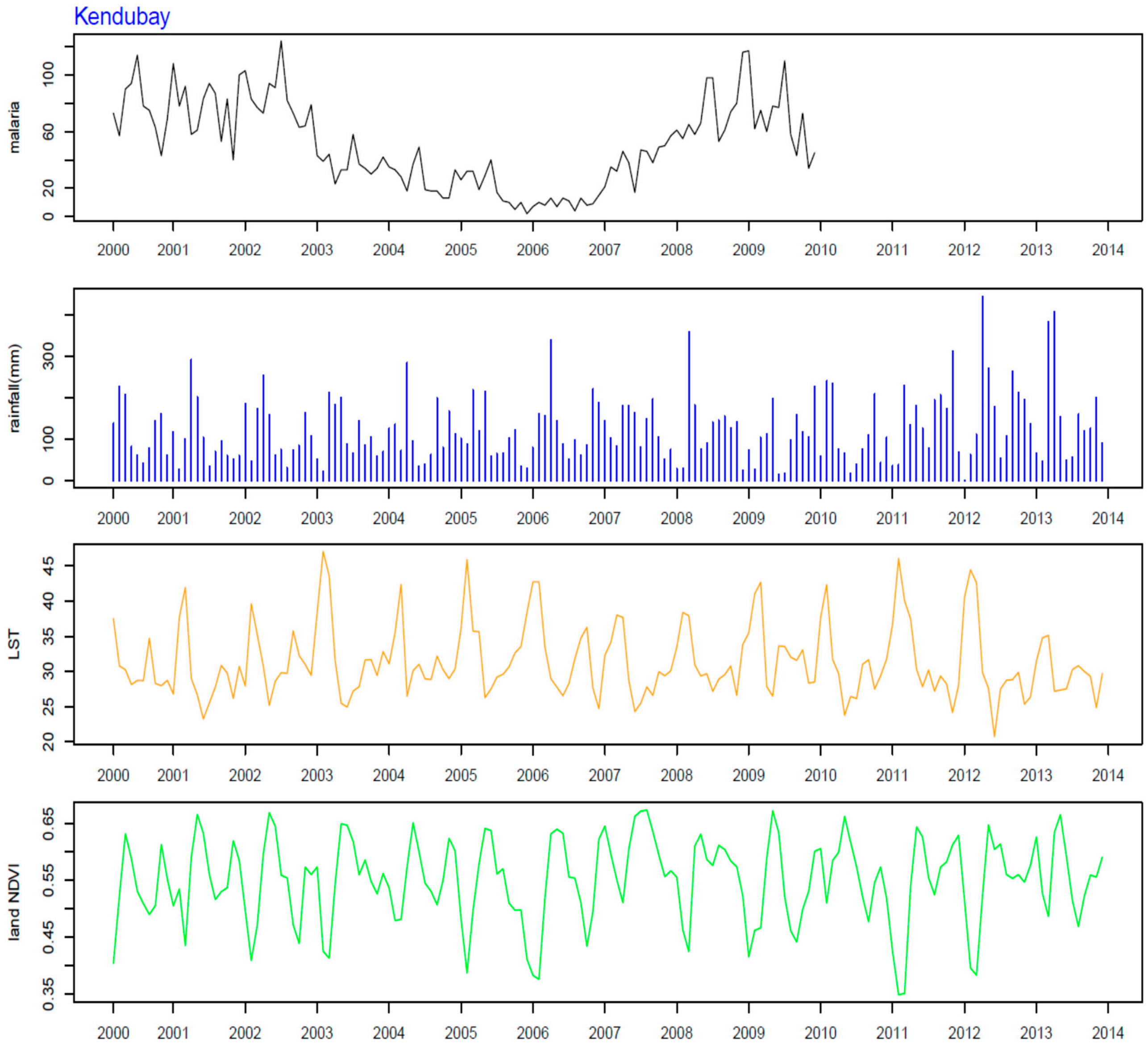

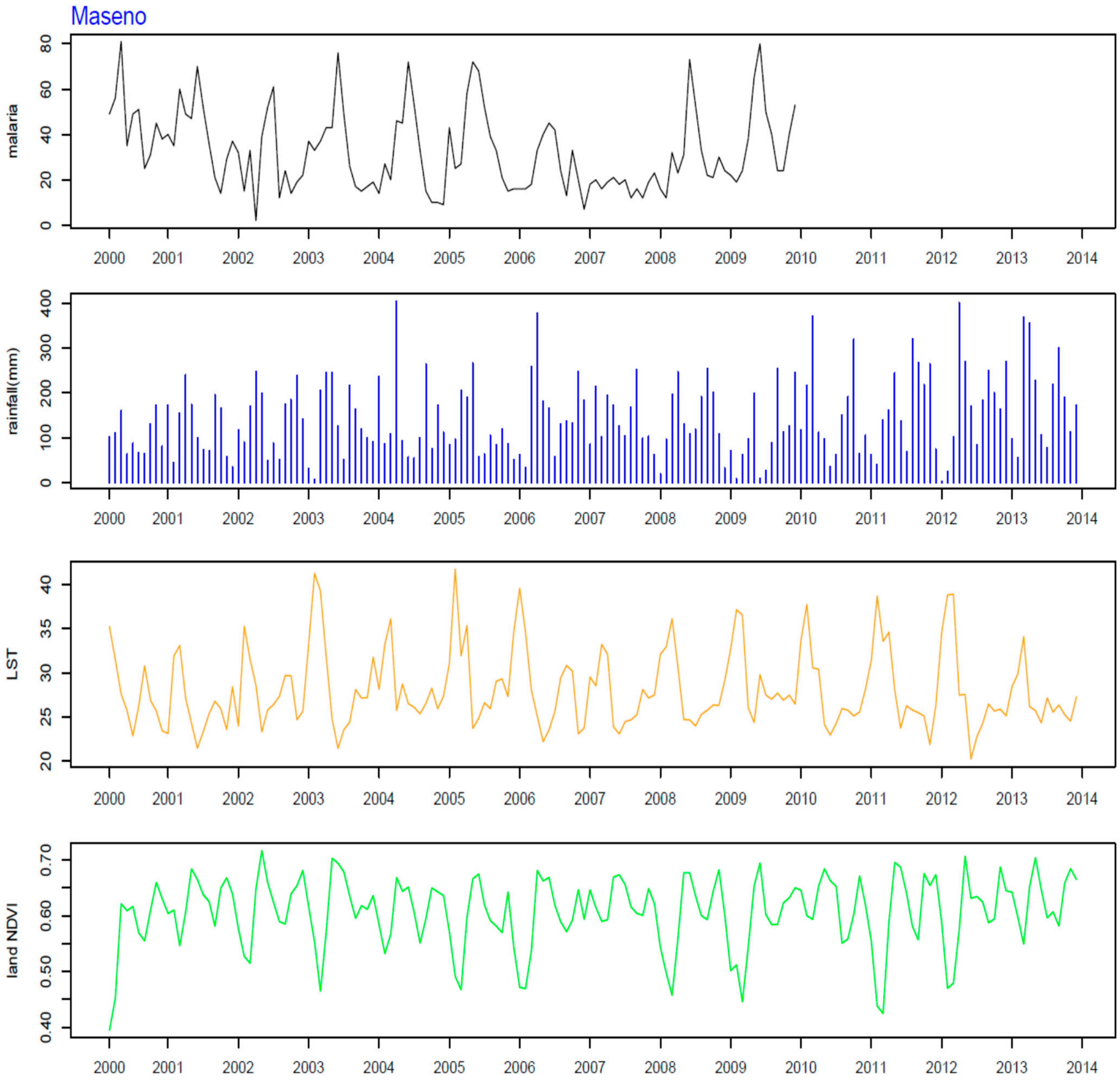
Maseno
malaria
rainfall(mm)
LST
land NDVI
0
20
40
60
80
100
200
300
400
25
30
35
40
0.40
0.50
0.60
0.70
2000
2001
2002
2003
2004
2005
2006
2007
2008
2009
2010
2011
2012
2013
2014

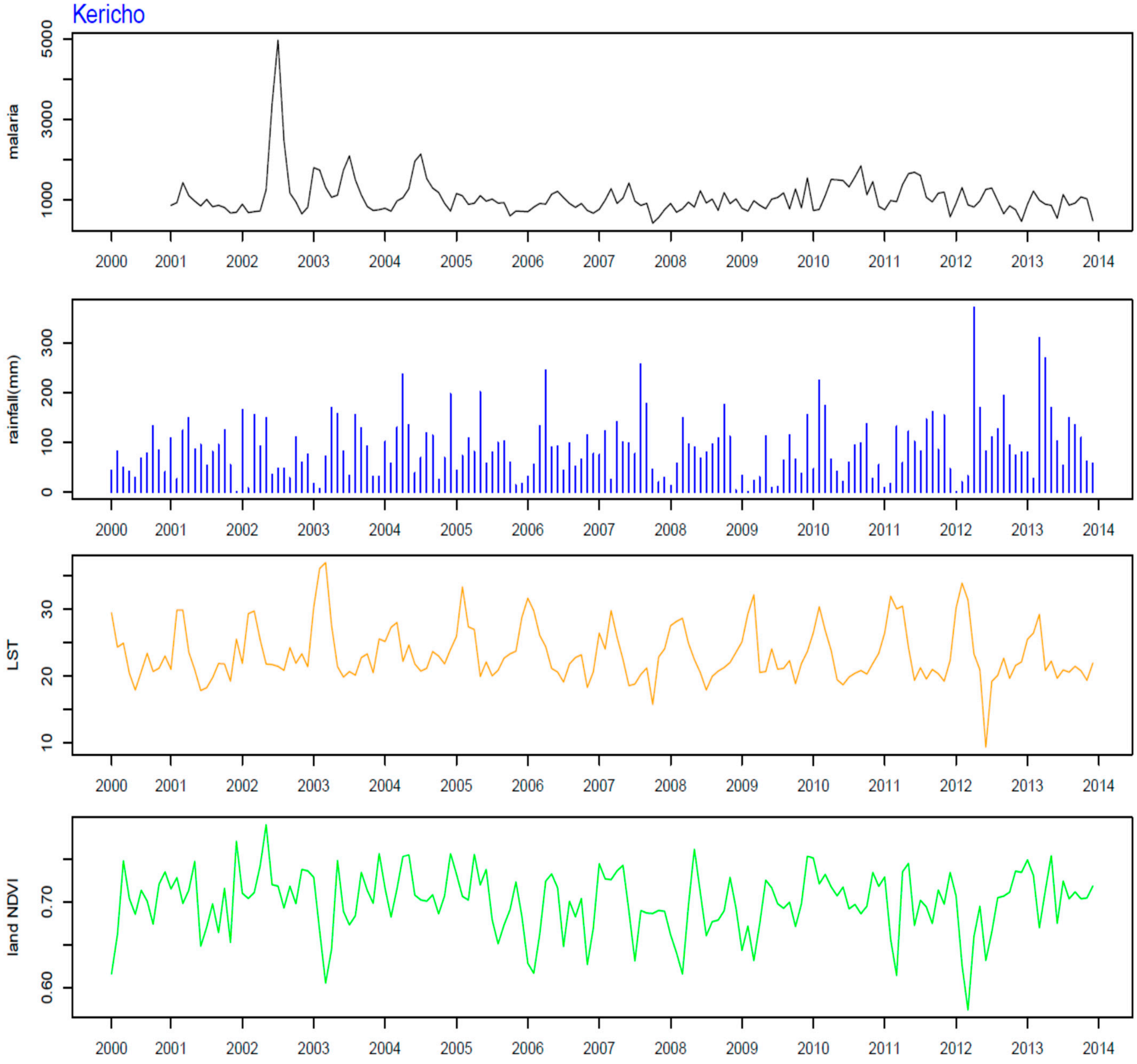
Kericho
malaria
1000
3000
5000
rainfall(mm)
0
100
200
300
LST
10
20
30
land NDVI
0.60
0.70
2000
2001
2002
2003
2004
2005
2006
2007
2008
2009
2010
2011
2012
2013
2014

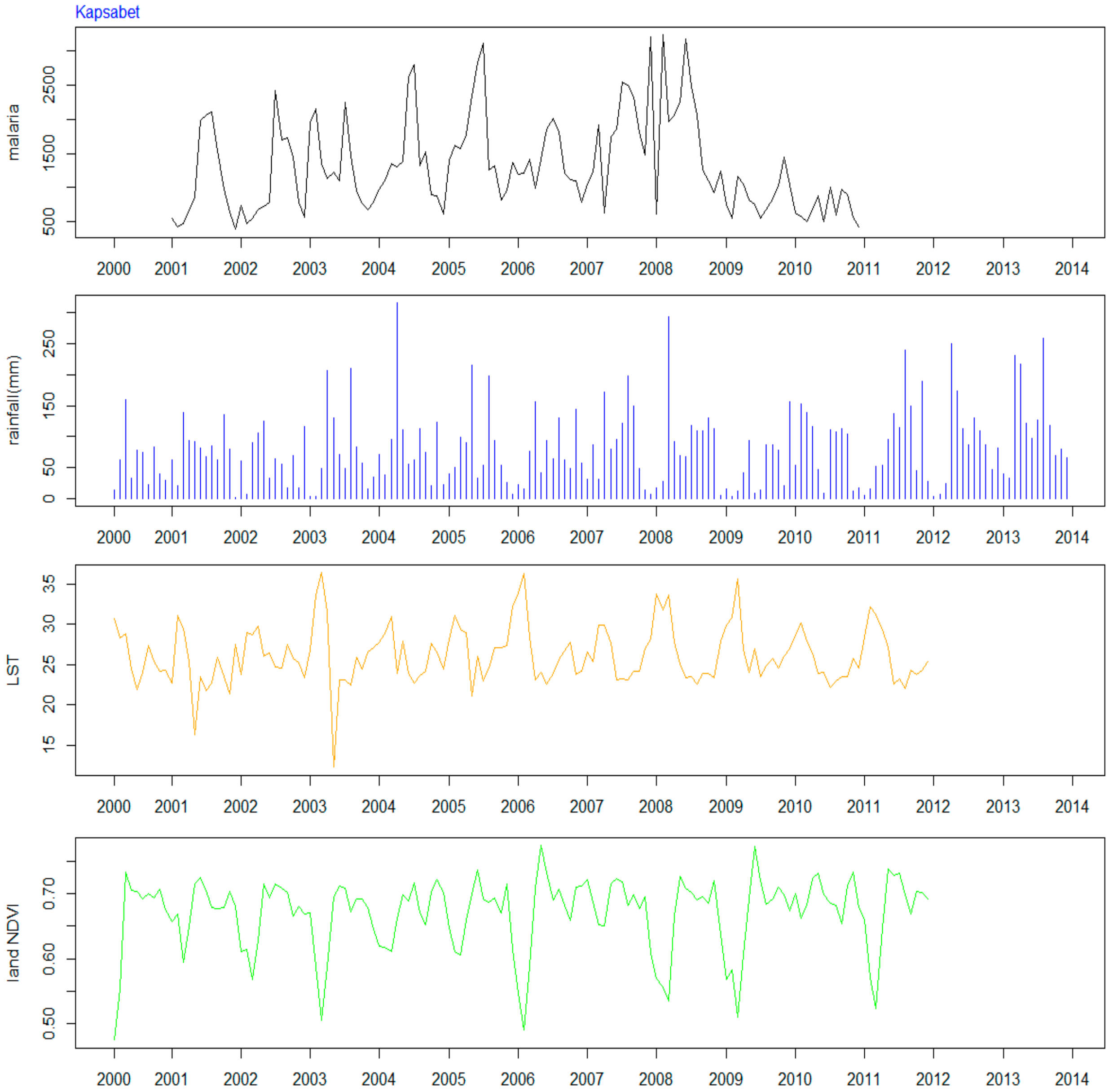
Kapsabet
malaria
500
1500
2500
rainfall(mm)
0
50
150
250
LST
15
20
25
30
35
land NDVI
0.50
0.60
0.70
2000
2001
2002
2003
2004
2005
2006
2007
2008
2009
2010
2011
2012
2013
2014

Figure S2 Associations between rainfall and monthly number of malaria cases in each hospital

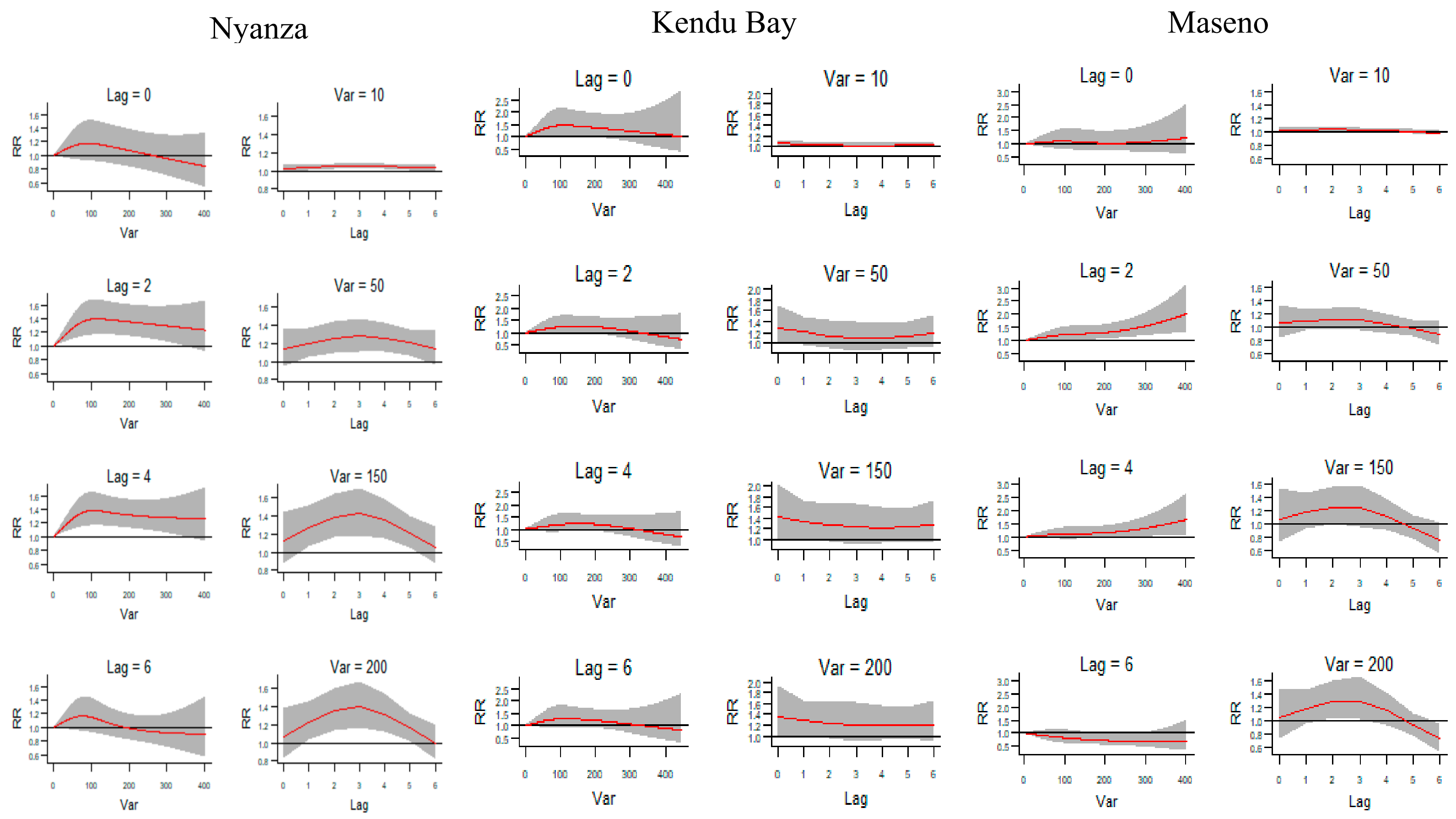

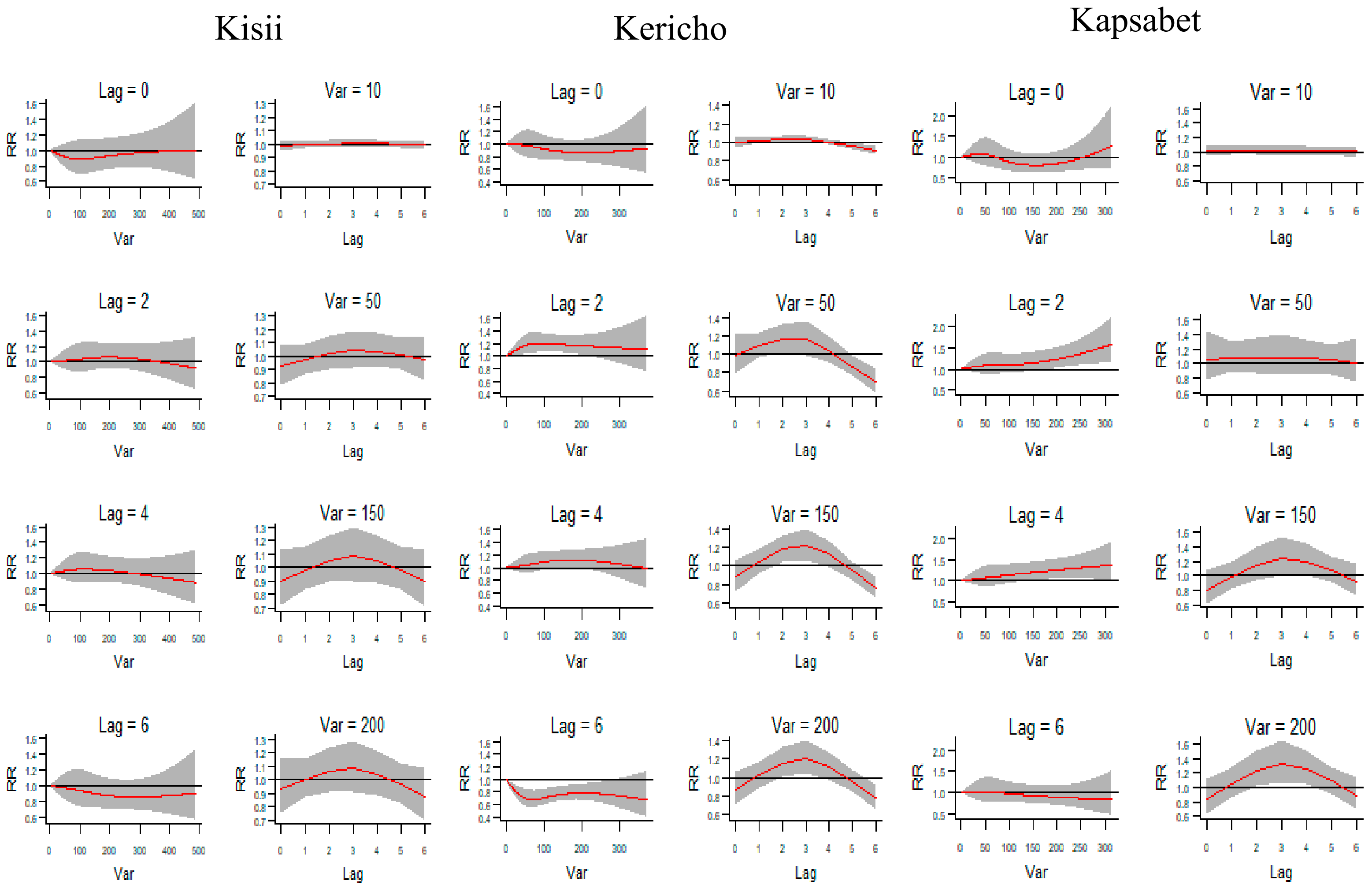
Kisii
Kericho
Kapsabet
Lag = 0
Var = 10
Lag = 0
Var = 10
Lag = 0
Var = 10
Lag = 2
Var = 50
Lag = 2
Var = 50
Lag = 2
Var = 50
Lag = 4
Var = 150
Lag = 4
Var = 150
Lag = 4
Var = 150
Lag = 6
Var = 200
Lag = 6
Var = 200
Lag = 6
Var = 200
RR
Var
Lag

Figure S3 Associations between LST and monthly number of malaria cases

(A) Pooled overall cumulative LST-malaria association

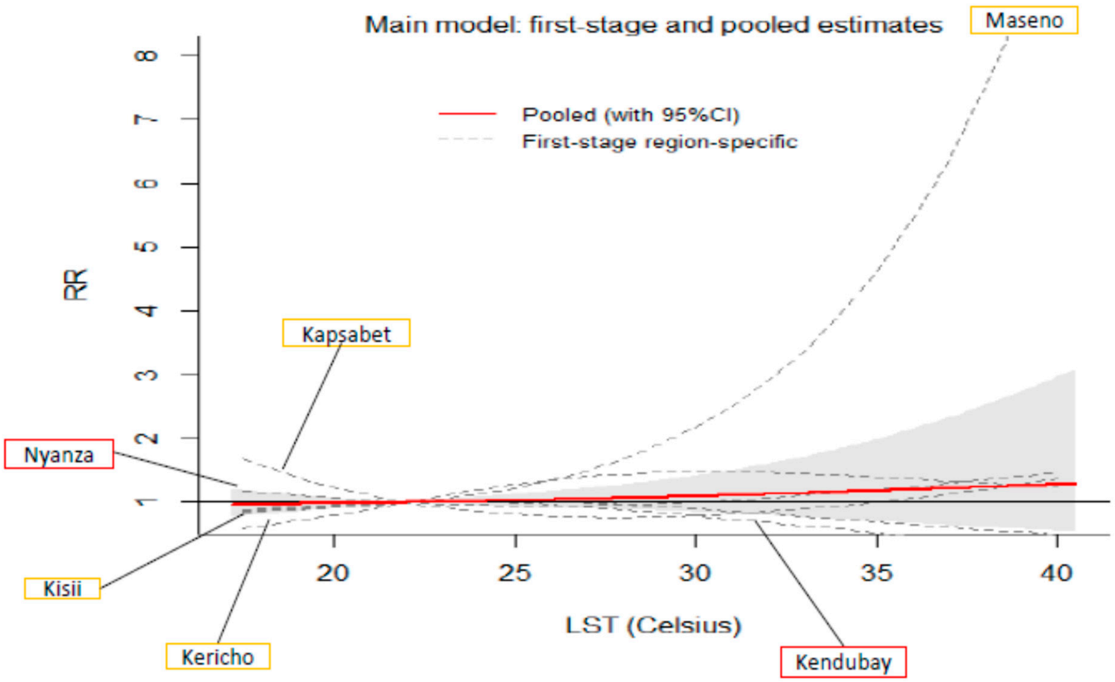

(B) Pooled predictor-specific LST-malaria association

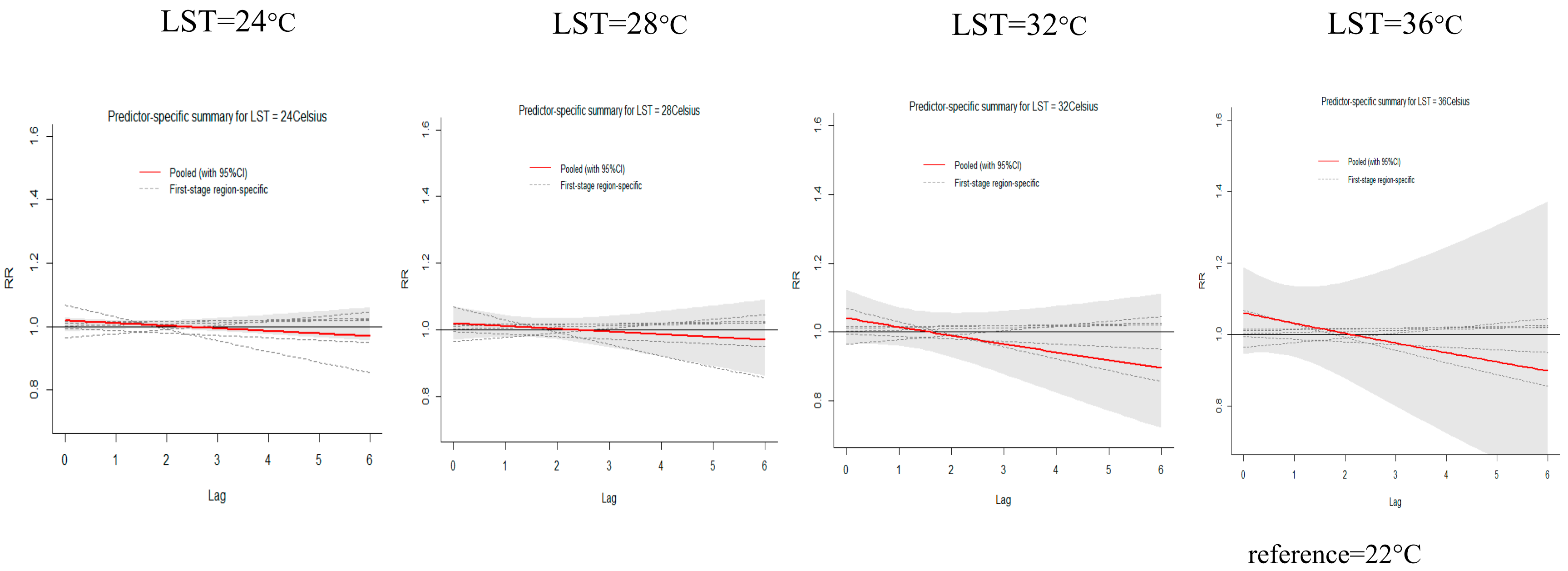

(C) LST-malaria association in each hospital

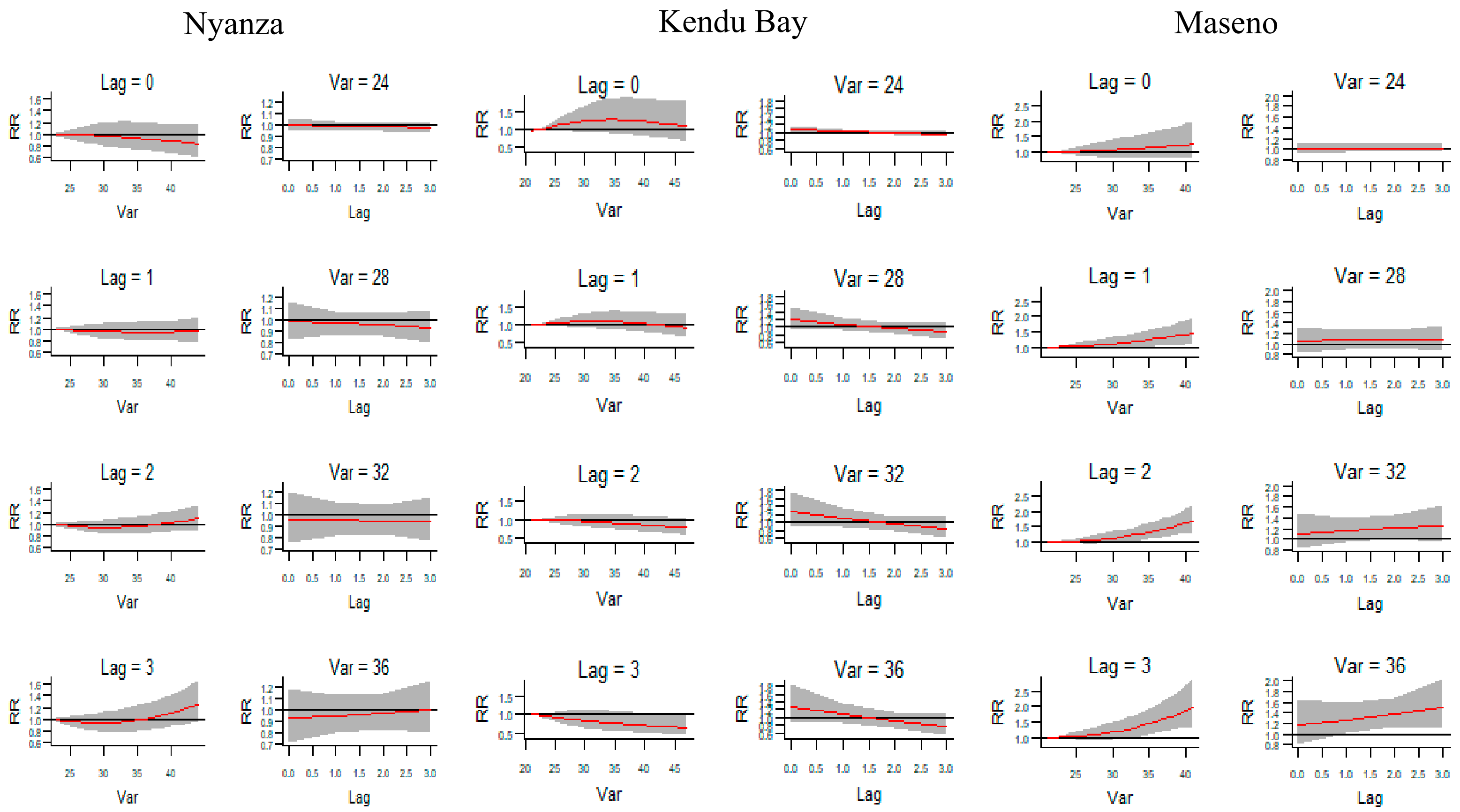

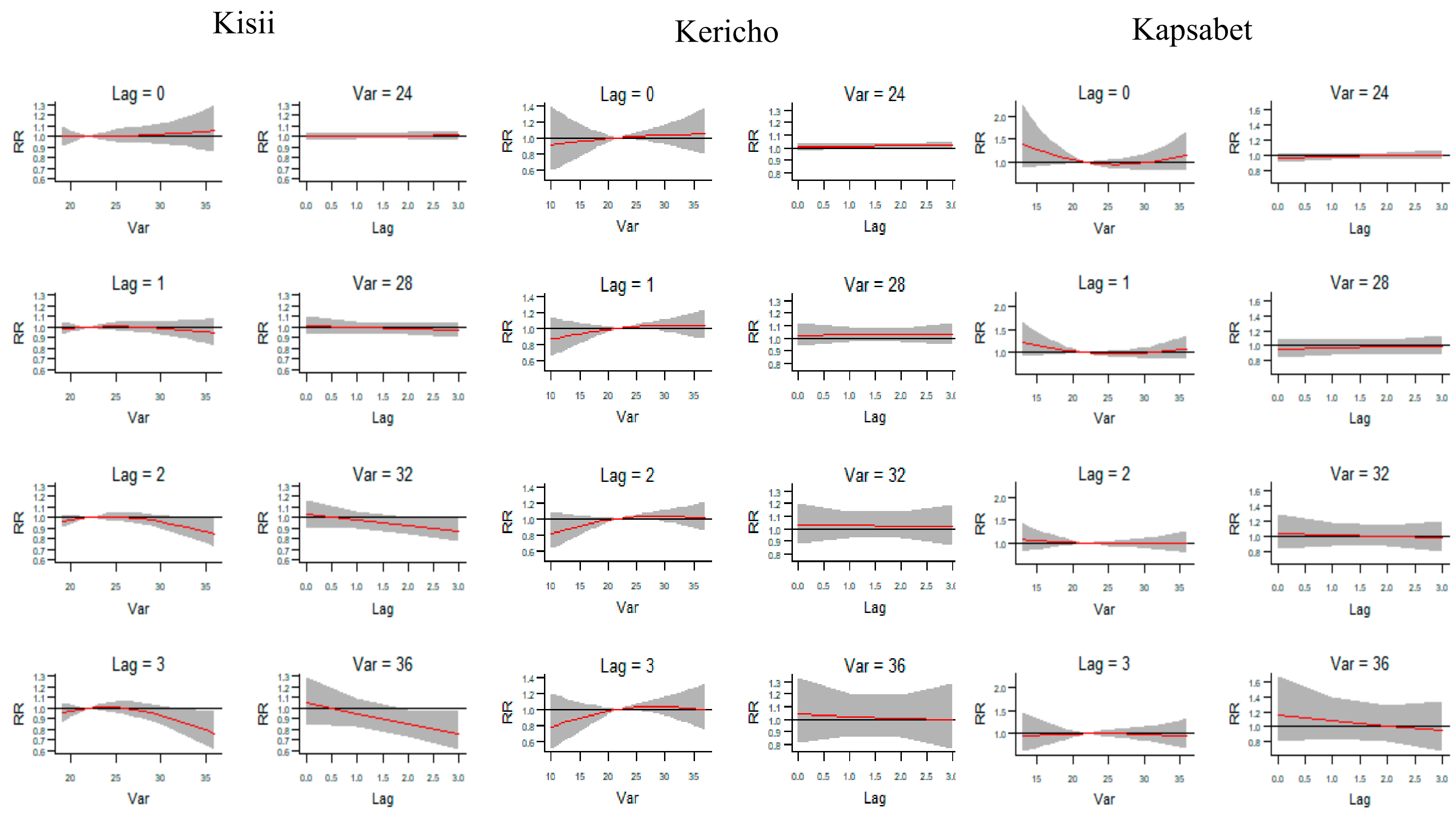

Kisii
Kericho
Kapsabet
Lag = 0
Var = 24
Lag = 1
Var = 28
Lag = 2
Var = 32
Lag = 3
Var = 36
RR
Var
Lag

Figure S4 Associations between rain and monthly number of malaria cases in lowland applying for Model 2 (the submodel)

(A) Pooled overall cumulative rainfall-malaria association

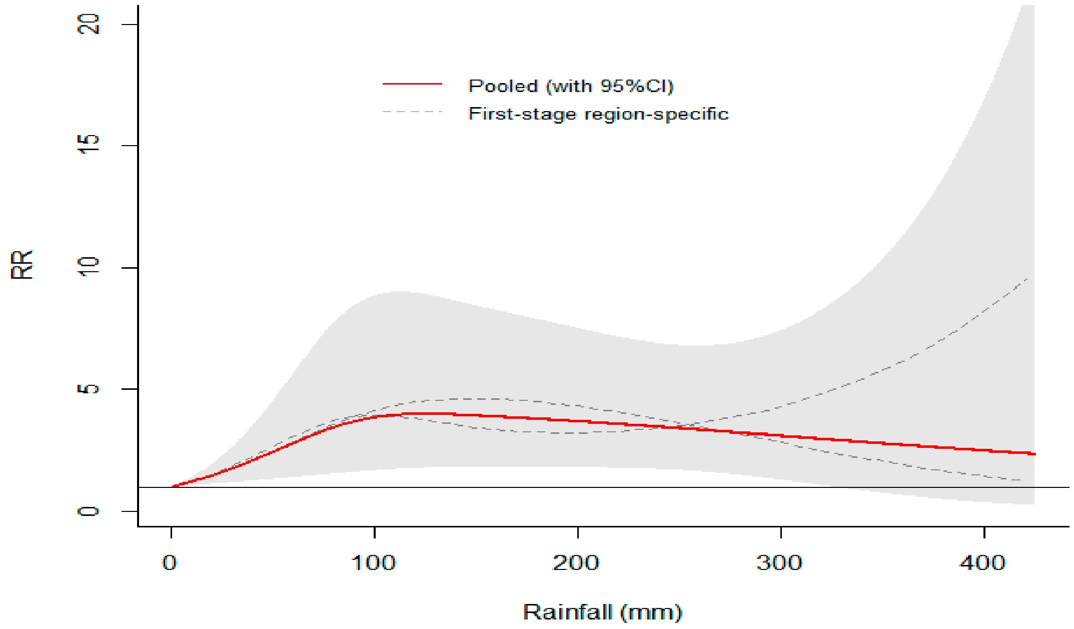

(B) Pooled predictor-specific rainfall-malaria association

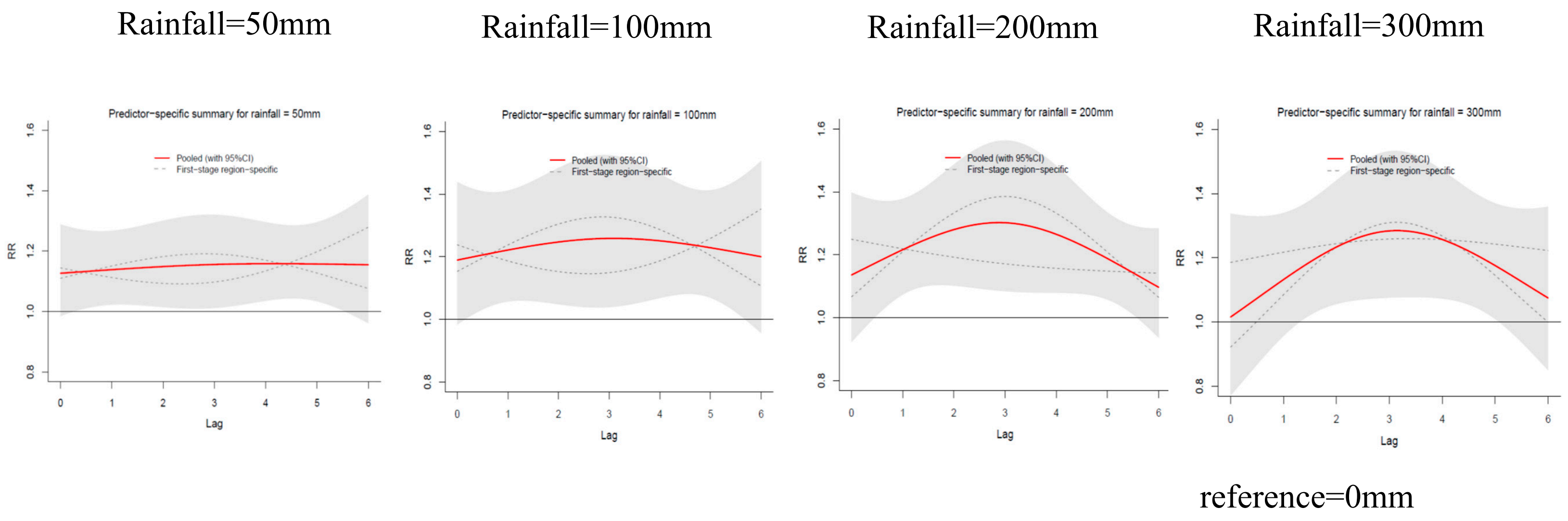

(C) rainfall-malaria association in each hospital

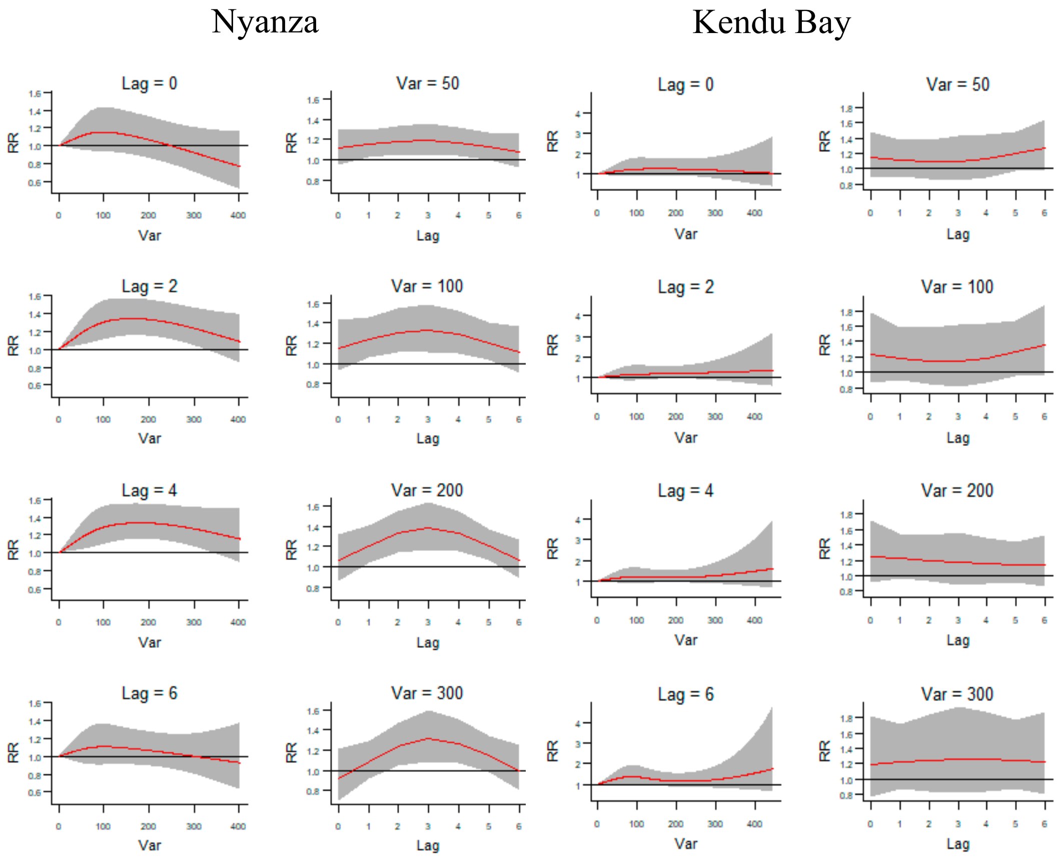

Figure S5 Associations between LST and monthly number of malaria cases lowland applying for Model 2 (the submodel)

(A) Pooled overall cumulative LST-malaria association

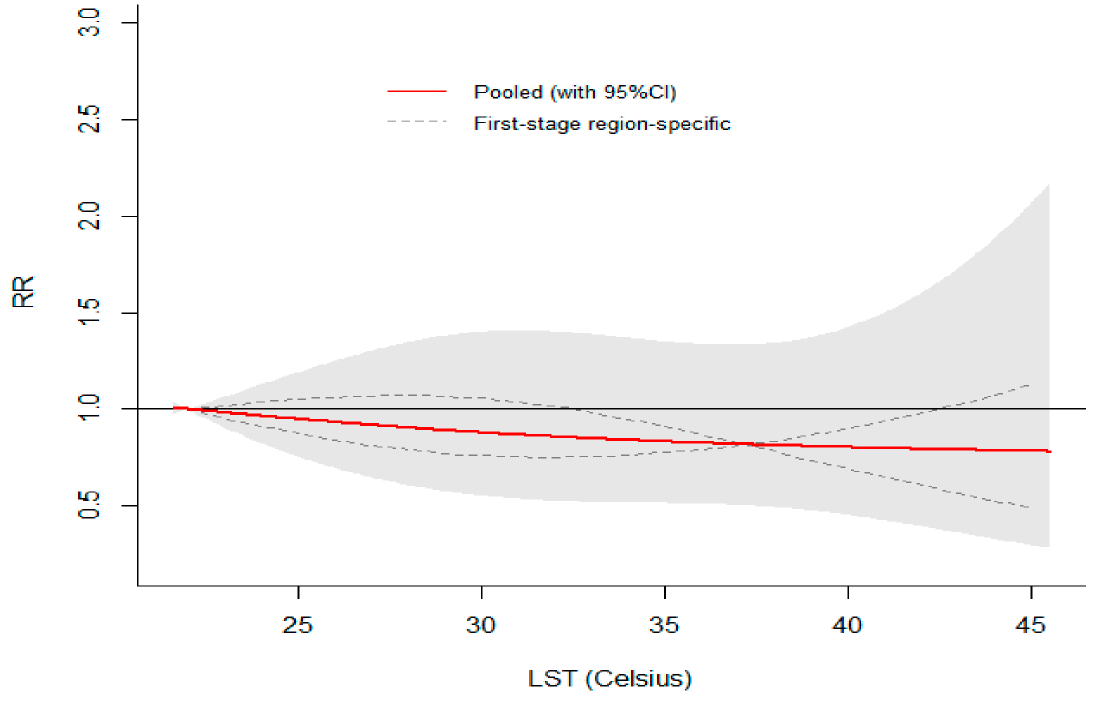

(B) Pooled predictor-specific LST-malaria association

LST=24°C LST=28°C LST=32°C LST=36°C

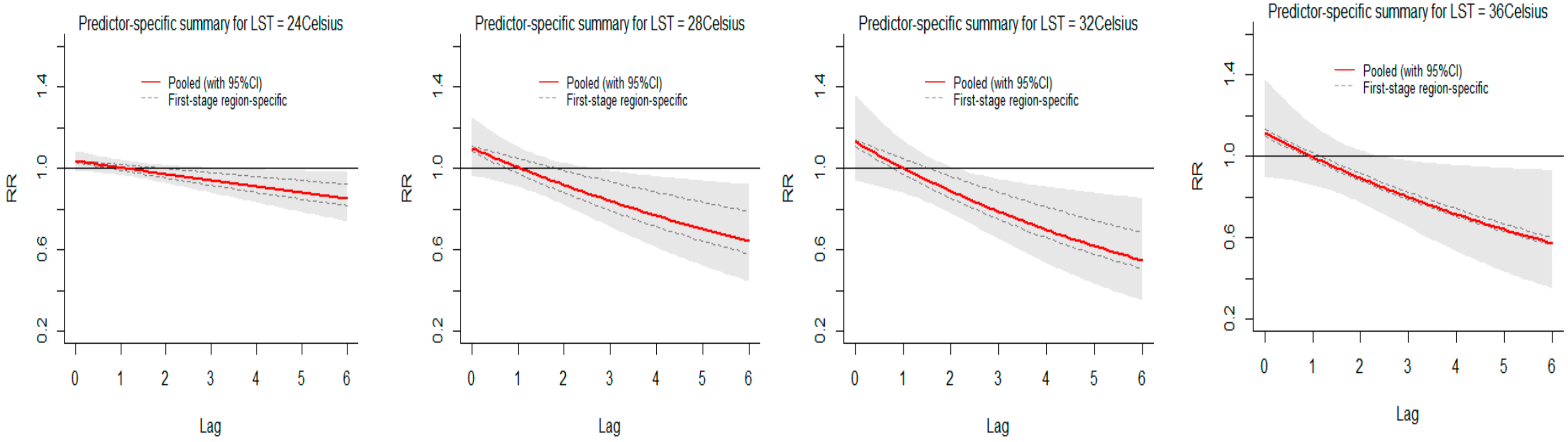

reference=22°C

Figure S6 Associations between LWL and monthly number of malaria cases in lowland applying for Model 2 (the submodel)

(A) Pooled overall cumulative LWL-malaria association

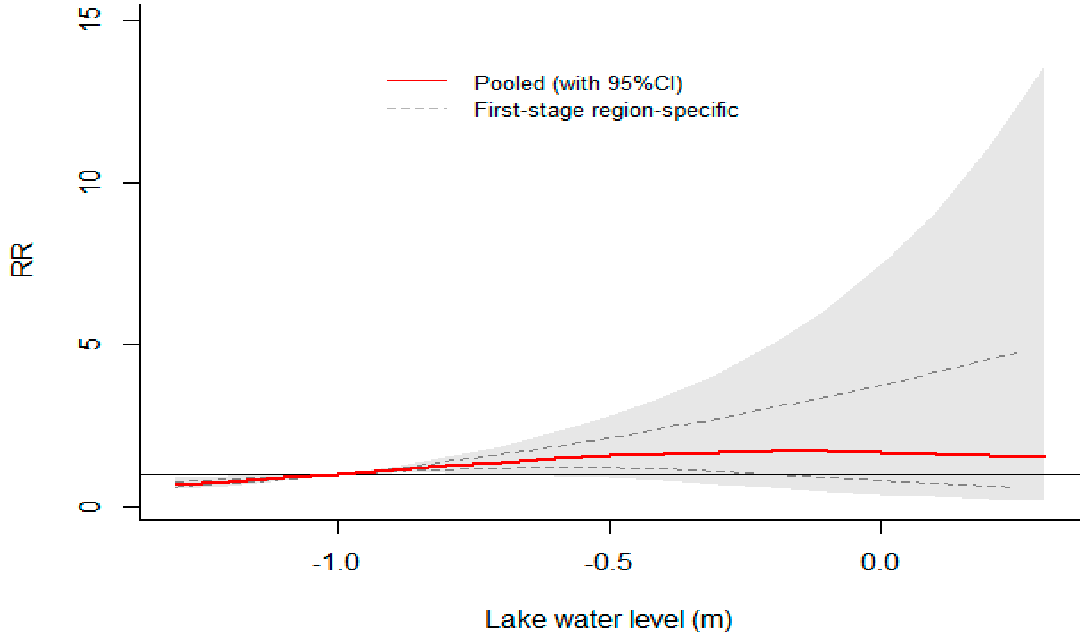

(B) Pooled predictor-specific LWL-malaria association

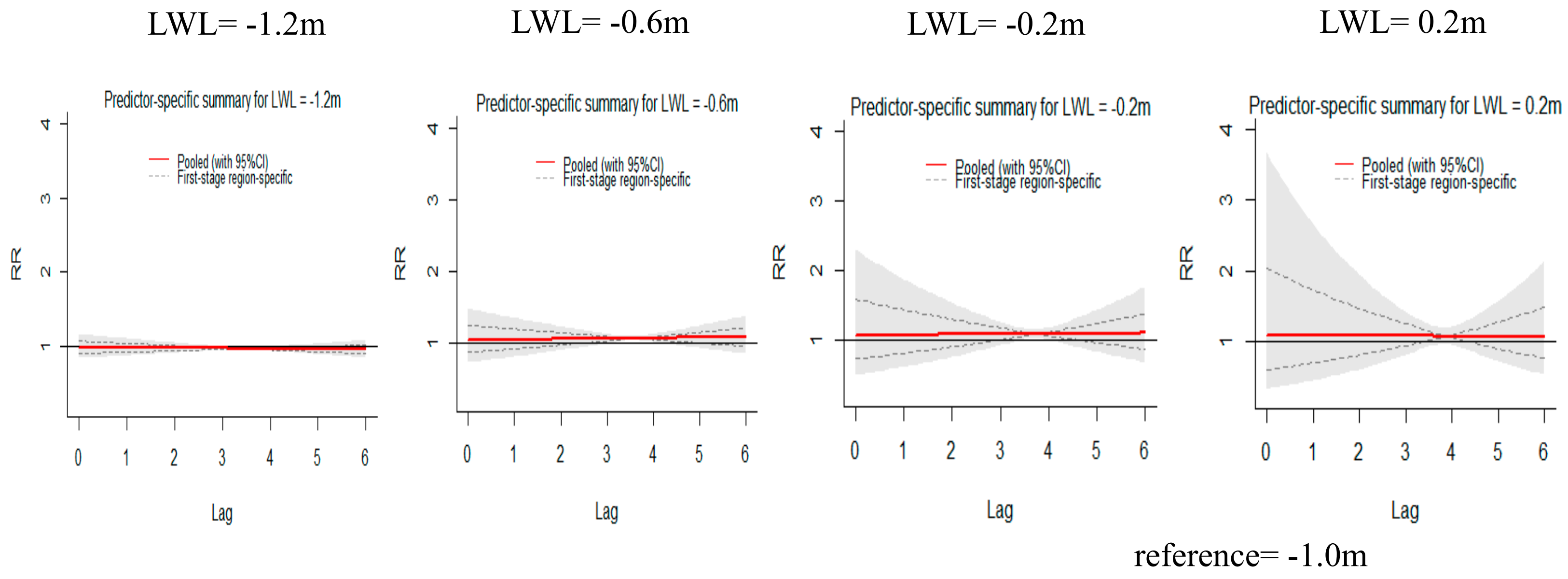

(C) LWL-malaria association in each hospital

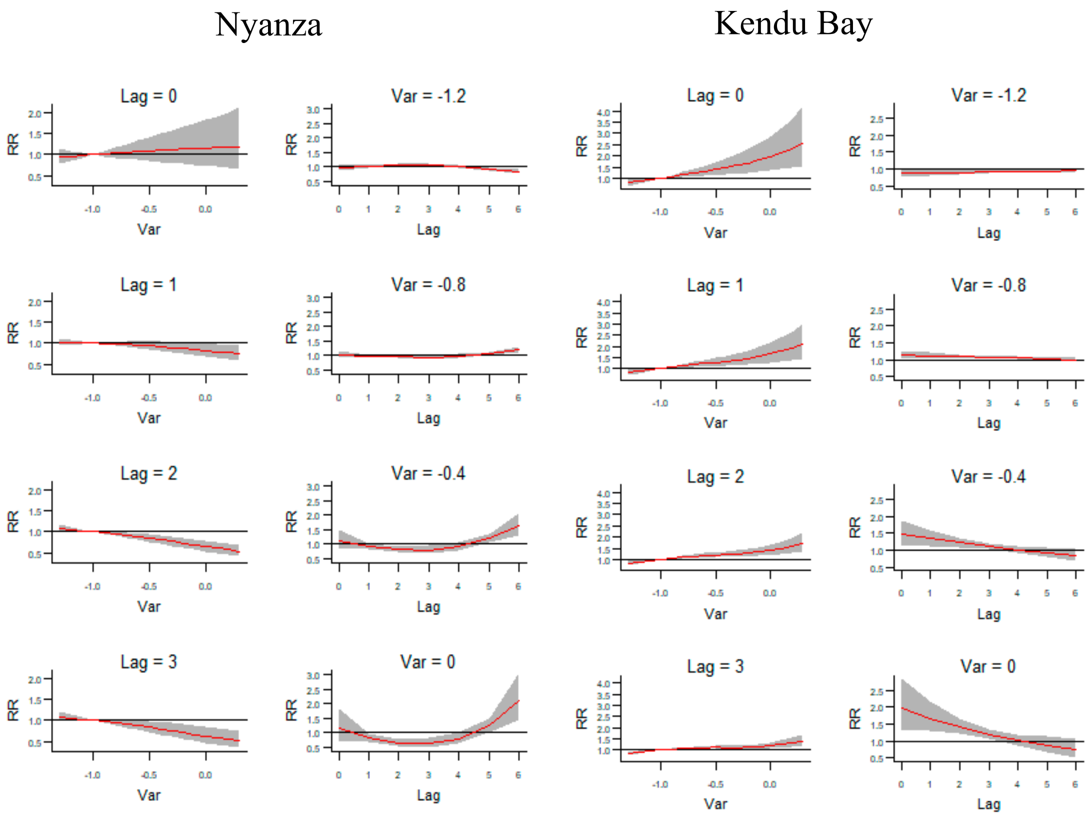

Table S1 pooled overall rainfall-malaria association by area (lowland & highland)

	lowland			highland		
rain[mm]	RR	95%CI		RR	95%CI	
10	1.29	1.12	1.48	1.03	0.96	1.10
50	3.35	1.73	6.49	1.14	0.81	1.59
100	6.89	2.55	18.62	1.21	0.74	1.97
120	7.32	2.74	19.56	1.20	0.74	1.93
150	6.92	2.81	17.02	1.17	0.76	1.80
200	5.72	2.51	13.04	1.16	0.78	1.73
300	3.49	1.29	9.45	1.31	0.77	2.24
400	1.92	0.38	9.75	1.67	0.64	4.36

Table S2 pooled lag-specific rainfall-malaria association by area (highland & lowland) in the model 1: the main model

lowland

	lag 0			lag 1			lag 2			lag 3			lag 4			lag 5			lag 6		
rain[mm]	RR	95%CI		RR	95%CI		RR	95%CI		RR	95%CI		RR	95%CI		RR	95%CI		RR	95%CI	
10	1.03	1.00	1.07	1.04	1.01	1.06	1.04	1.02	1.07	1.04	1.02	1.07	1.04	1.02	1.07	1.03	1.01	1.06	1.03	0.99	1.07
50	1.17	1.00	1.37	1.20	1.06	1.34	1.22	1.08	1.37	1.22	1.08	1.39	1.21	1.09	1.36	1.17	1.04	1.32	1.14	0.94	1.37
100	1.28	1.01	1.62	1.34	1.12	1.59	1.38	1.15	1.65	1.39	1.15	1.69	1.37	1.15	1.62	1.28	1.07	1.53	1.21	0.92	1.58
120	1.29	1.02	1.63	1.35	1.13	1.61	1.40	1.17	1.68	1.42	1.16	1.72	1.39	1.17	1.65	1.29	1.09	1.53	1.20	0.92	1.55
150	1.27	1.01	1.59	1.35	1.14	1.59	1.40	1.18	1.67	1.42	1.18	1.71	1.38	1.18	1.63	1.27	1.10	1.48	1.16	0.92	1.46
200	1.22	0.98	1.53	1.32	1.13	1.53	1.39	1.18	1.64	1.41	1.17	1.69	1.36	1.16	1.59	1.24	1.09	1.41	1.10	0.91	1.34
300	1.11	0.87	1.42	1.23	1.03	1.46	1.31	1.05	1.64	1.35	1.05	1.73	1.30	1.06	1.59	1.16	1.01	1.34	1.01	0.82	1.23
400	1.00	0.73	1.37	1.12	0.87	1.44	1.21	0.84	1.74	1.26	0.84	1.88	1.22	0.89	1.68	1.08	0.87	1.35	0.94	0.69	1.28

highland

	lag 0			lag 1			lag 2			lag 3			lag 4			lag 5			lag 6		
rain[mm]	RR	95%CI		RR	95%CI		RR	95%CI		RR	95%CI		RR	95%CI		RR	95%CI		RR	95%CI	
10	1.00	0.98	1.02	1.01	1.00	1.03	1.02	1.01	1.03	1.02	1.00	1.04	1.01	1.00	1.02	0.99	0.98	1.01	0.98	0.95	1.01
50	0.99	0.90	1.10	1.05	0.99	1.13	1.09	1.03	1.17	1.10	1.02	1.18	1.05	0.99	1.12	0.98	0.91	1.05	0.91	0.80	1.03
100	0.97	0.84	1.13	1.08	0.98	1.19	1.16	1.05	1.28	1.17	1.05	1.31	1.09	0.99	1.21	0.96	0.87	1.07	0.85	0.71	1.01
120	0.96	0.83	1.11	1.08	0.98	1.18	1.17	1.06	1.29	1.19	1.06	1.33	1.10	1.00	1.21	0.96	0.87	1.06	0.83	0.70	0.99
150	0.93	0.82	1.07	1.07	0.98	1.16	1.17	1.07	1.28	1.20	1.07	1.33	1.11	1.01	1.21	0.96	0.88	1.05	0.83	0.71	0.96
200	0.91	0.80	1.04	1.06	0.97	1.15	1.18	1.08	1.29	1.21	1.09	1.34	1.12	1.03	1.22	0.97	0.90	1.04	0.82	0.72	0.92
300	0.92	0.80	1.06	1.08	0.98	1.18	1.21	1.07	1.38	1.25	1.08	1.45	1.15	1.02	1.29	0.97	0.89	1.05	0.80	0.71	0.91
400	0.98	0.81	1.19	1.14	0.98	1.32	1.27	1.02	1.59	1.30	1.02	1.67	1.19	0.98	1.44	0.97	0.85	1.11	0.80	0.66	0.97

Table S3 pooled overall rainfall-malaria association by area (lowland & highland) with different rainfall lag and DF for month of year

(A) for different rainfall lag (lag=4 to 8)

Lowland

	lag=4				lag=5				lag=6(main model)				lag=7				lag=8		
rain [mm]	RR	95%CI			RR	95%CI			RR	95%CI			RR	95%CI			RR	95%CI	
10	1.17	1.05	1.29	10	1.23	1.08	1.39	10	1.29	1.12	1.48	10	1.39	1.20	1.61	10	1.42	1.21	1.68
100	3.31	1.59	6.89	100	4.86	1.96	12.05	100	6.89	2.55	18.62	100	11.29	3.94	32.29	100	13.29	4.09	43.13
120	3.52	1.68	7.35	120	5.22	2.12	12.88	120	7.32	2.74	19.56	120	11.47	4.03	32.68	120	13.40	4.18	43.00
200	3.34	1.69	6.58	200	4.58	2.06	10.16	200	5.72	2.51	13.04	200	6.78	2.81	16.37	200	7.24	2.77	18.93
300	2.61	1.04	6.57	300	2.99	1.01	8.81	300	3.49	1.29	9.45	300	3.68	1.42	9.54	300	3.47	1.23	9.83
400	1.81	0.39	8.45	400	1.64	0.26	10.39	400	1.92	0.38	9.75	400	2.17	0.59	7.97	400	1.80	0.44	7.43

Highland

	lag=4				lag=5				lag=6(main model)				lag=7				lag=8		
rain [mm]	RR	95%CI			RR	95%CI			RR	95%CI			RR	95%CI			RR	95%CI	
10	1.06	1.00	1.12	10	1.05	0.98	1.12	10	1.03	0.96	1.10	10	1.03	0.96	1.10	10	1.04	0.96	1.12
100	1.54	1.04	2.31	100	1.39	0.88	2.21	100	1.21	0.74	1.97	100	1.14	0.71	1.84	100	1.22	0.72	2.04
120	1.55	1.04	2.31	120	1.39	0.89	2.19	120	1.20	0.74	1.93	120	1.10	0.69	1.75	120	1.16	0.69	1.93

200	1.51	1.05	2.17	200	1.35	0.90	2.02	200	1.16	0.78	1.73	200	0.96	0.64	1.43	200	0.91	0.59	1.42
300	1.71	1.00	2.92	300	1.47	0.80	2.72	300	1.31	0.77	2.24	300	1.09	0.68	1.76	300	0.83	0.49	1.40
400	2.20	0.86	5.63	400	1.78	0.58	5.47	400	1.67	0.64	4.36	400	1.55	0.73	3.30	400	0.88	0.38	2.04

(B) for different month of year DF (mdf)

mdf (Nyanza, Maseno, Kendubay, Kapsabet, Kericho, Kisii)

Lowland

	**(main model)**														
**mdf**	**(4,4,4,2,2,2)**				**(2,2,2,2,2,2)**				**(3,3,3,3,3,3)**				**(4,4,4,4,4,4)**		
rain [mm]	RR	95%CI			RR	95%CI			RR	95%CI			RR	95%CI	
10	1.29	1.12	1.48	10	1.29	1.13	1.48	10	1.26	1.10	1.45	10	1.29	1.12	1.48
100	6.89	2.55	18.62	100	6.93	2.67	17.97	100	5.82	2.21	15.34	100	6.95	2.58	18.75
120	7.32	2.74	19.56	120	7.25	2.82	18.61	120	6.10	2.34	15.91	120	7.39	2.77	19.69
200	5.72	2.51	13.04	200	5.44	2.47	11.96	200	4.73	2.11	10.57	200	5.76	2.53	13.13
300	3.49	1.29	9.45	300	3.56	1.40	9.01	300	3.05	1.18	7.88	300	3.56	1.34	9.46
400	1.92	0.38	9.75	400	2.30	0.53	10.01	400	1.87	0.42	8.26	400	2.00	0.42	9.53

Highland

	(main model)														
**mdf**	**(4,4,4,2,2,2)**				**(2,2,2,2,2,2)**				**(3,3,3,3,3,3)**				**(4,4,4,4,4,4)**		
rain [mm]	RR	95%CI			RR	95%CI			RR	95%CI			RR	95%CI	
10	1.03	0.96	1.10	10	1.03	0.96	1.10	10	1.02	0.96	1.10	10	1.03	0.96	1.11
100	1.21	0.74	1.97	100	1.19	0.74	1.91	100	1.18	0.73	1.91	100	1.28	0.78	2.09
120	1.20	0.74	1.93	120	1.18	0.75	1.87	120	1.17	0.73	1.87	120	1.28	0.79	2.06
200	1.16	0.78	1.73	200	1.15	0.77	1.70	200	1.13	0.76	1.68	200	1.27	0.84	1.91
300	1.31	0.77	2.24	300	1.29	0.77	2.15	300	1.28	0.76	2.14	300	1.49	0.87	2.55
400	1.67	0.64	4.36	400	1.64	0.68	3.95	400	1.63	0.67	3.96	400	1.98	0.77	5.04
